# Enhancing public health intelligence workforce capacity and capability: insights from English local authorities’ COVID-19 response

**DOI:** 10.3389/fpubh.2026.1833664

**Published:** 2026-07-01

**Authors:** Janette Parr, Yen-Fu Chen, Amy Grove

**Affiliations:** 1 Centre for Evidence and Implementation Science, School of Social Policy and Society, The University of Birmingham, Birmingham, United Kingdom; 2 Department of Health and Welfare, College of City Management, University of Taipei, Taipei, Taiwan

**Keywords:** case studies, disease outbreak, integration, local government, mixed methods, public health informatics, SARS-CoV-2, survey

## Abstract

**Introduction:**

The COVID-19 pandemic provided a stimulus to improve emergency response capabilities in the United Kingdom. Compared to healthcare workers, there is a paucity of research examining the experiences of public health staff involved in the response. During the pandemic, English local authorities (LAs) responded to limit virus spread and mitigate its impacts by providing public health intelligence (PHI). This article reports workforce-related findings from a larger study which focused on understanding sub-national provision of PHI during an infectious disease emergency.

**Methods:**

A mixed method, sequential, exploratory study was conducted. This involved comparative case studies of LA responses and an anonymous online survey of LA PHI staff followed by data integration. The primary data collection method for the case studies was semi-structured interviews with PHI staff. A cross-case analysis of interview data using inductive thematic analysis was performed. Quantitative survey data was analysed to produce descriptive statistics and long-form text responses supplemented the interview data. Qualitative and quantitative data were integrated by using case study findings to inform survey topics and by following an established framework to examine confirmation, complementarity, expansion, or discordance.

**Results:**

Data from 12 interviews and 120 survey responses was integrated. The pandemic saw changing demands for analytical capacity—the highest demand occurring at the outset. Inequities in allocation of keyworker status impacted those with childcare responsibilities. Staff identified knowledge gaps around health protection, data automation, information governance, and emerging data sources. PHI personnel had traumatic experience, and staff well-being was adversely affected by high workloads, limited leave, exposure to emotive data, and difficulties in disconnecting. Anticipating pandemics and emotional preparedness were valued mindsets. Peer support and effective leadership mitigated negative effects on wellbeing.

**Discussion:**

The traumatic effect of responding to the pandemic on some PHI staff might not be anticipated in a group not considered “frontline”. We propose there is a duty to prepare staff for emergencies and implement best human resource practices. Findings suggest a need to improve public health workforce planning for emergencies. Emergency response plans should include the PHI function and be tied to public health authorities’ capacity and capability to enact them.

## 1 Introduction

The first SARS-CoV-2 cases were detected in China in December 2019. Subsequently, the virus spread rapidly, and the World Health Organization (WHO) characterised the outbreak as a pandemic on 11th March 2020 (1). Up to June 2023, the number of United Kingdom deaths involving COVID-19 was estimated to be over 225,000 (2). In England, Directors of Public Health (DPH) are statutory chief officers based in local authorities (LAs). They are responsible for delivering public health duties and have a system leadership role to improve public health across their region (3). Throughout the COVID-19 pandemic, DPH and their teams responded to limit the spread of the virus and to mitigate its wider impacts. Provision of public health intelligence (PHI) was an important component of response (4). This study defines PHI as a function that supports the protection and improvement of the public’s health by directing, gathering, analysing, interpreting, and disseminating data and information.

This paper presents findings from a PhD project examining factors influencing PHI provision by LAs during the COVID-19 pandemic (5). By exploring the views and experiences of PHI staff, our mixed methods study aimed to identify ways to enhance policy and practice to improve preparedness for future infectious disease emergencies. Interviews with PHI staff involved in the COVID-19 response identified several key elements shaping it, including data infrastructure, public support, inter-organisational collaboration, leadership and management, and workforce capacity and capability. Among these factors, PHI workforce capacity and capability emerged as critical to the response, and this article focuses specifically on this aspect. Our study frames workforce capacity as the total human resource available for deployment, and workforce capability as the dynamic combination of individual competencies and organisational factors (e.g., technology, culture, and leadership) that empower employees to function effectively within their roles. The WHO has defined competencies as: “*The abilities of a person to integrate knowledge, skills and attitudes in their performance of tasks in a given context*” (6). The context in which staff work includes factors such as processes, technology, equity, job satisfaction, team diversity, and management which collectively influence workforce performance (7).

In England, staff involved in the provision of PHI work in many different organisations and have widely varying job descriptions and titles. The Faculty of Public Health have distinguished three categories of PHI roles, namely, (1) PHI specialists, (2) a broader group of PHI practitioners, and (3) the many public health specialists with an intelligence component within their overall responsibilities (8). Many personnel delivering PHI at a sub-national level work in LAs. Prior to the COVID-19 pandemic, multiple re-organisations and budget cuts were a challenge for the public health system (9, 10). Additionally, The Centre for Workforce Intelligence (CfWI) had raised concerns around the PHI workforce, highlighting uncertainty around existing numbers of posts, lack of career progression and workforce mobility, and inadequate national support for local PHI teams (11). The information and knowledge management carried out by PHI personnel is essential for effective health emergency and disaster risk management. The WHO strongly supports careful planning, adequate staffing, and thorough training to strengthen this function (12). However, we currently lack a clear understanding of what effective workforce planning for emergency situations entails and what might help for the future. Whether the PHI workforce’s capacity and capability were sufficient is an important and under-explored part of the COVID-19 response (most previous work has concerned clinical staff).

Therefore, exploring the contribution of the PHI function to the COVID-19 response, and what facilitated or challenged this, is essential. This information is valuable to inform more appropriate public health emergency preparedness (PHEP) which may help to reduce morbidity and mortality from future pandemics and mitigate against their inequitable impacts (2).

## 2 Materials and methods

**Figure 1:**
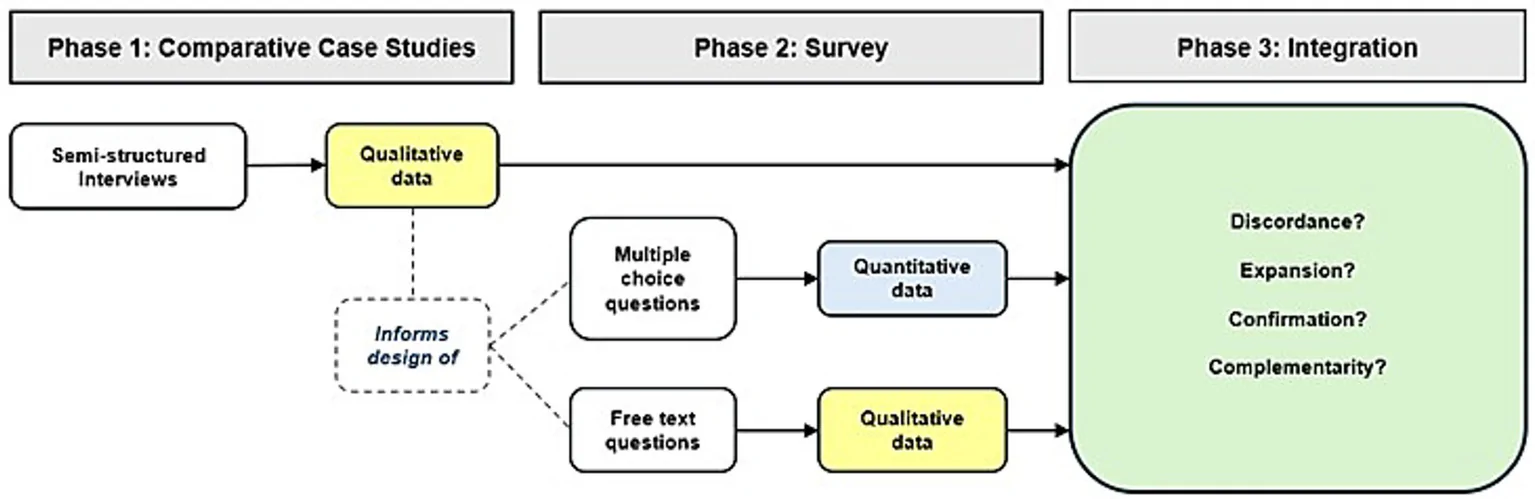
Study design.

This was a mixed methods, sequential, exploratory study as described by Curry and Nunez-Smith (13). There were three phases to data collection and analysis: (1) Comparative case studies investigating public health intelligence (PHI) provision by local authorities (LAs) during the COVID-19 pandemic; (2) An anonymous online survey of LA PHI staff involved in the response; (3) Qualitative and quantitative data was integrated using a method which considers confirmation, complementarity, expansion, or discordance between different data types (14). Figure 1 shows the study design.

### 2.1 Sampling and recruitment

In keeping with the study’s exploratory aims, all English LAs were eligible to take part as case studies and recruitment of LAs was opportunistic (aiming for a minimum of three). A variety of means were used to recruit sites, including: emails to senior staff working in West Midlands LAs (including Directors of Public Health, Public Health Consultants and Public Health Managers), a presentation to a professional network—the Midlands Intelligence Network (MiNet), and messages sent via a public sector networking website (Knowledge Hub) to regional and national Office for Health Improvement and Disparities (OHID) PHI groups. All LAs that expressed an interest were included. Individuals’ eligibility to participate was the same for the case studies and the survey. The eligibility criteria were:

1. Employed by [the LA/a LA] and have been so for a period of at least 3 months.
2. Currently, or have previously been, engaged in COVID-19 related PHI activity between January 2020 and present for [the LA/a LA].

PHI activity was defined for participants as “*activity funded by a public health grant and/or directed by a United Kingdom Public Health Register (UKPHR) registered professional (for example, a Public Health Consultant) including the direction, gathering, analysis, interpretation and dissemination of data and information including research evidence*.”

A link to the online survey was provided by email to a variety of organisations who were asked to distribute it to relevant personnel. Organisations that distributed the survey included: Association of the Directors of Public Health (ADPH); JISC Mailing Lists: PHI and Public Health; Local Area Research and Intelligence Association (LARIA); LAs—LAs with Directors of Public Health were identified and their public health/intelligence teams emailed directly; Local Government Association (LGA); National Institute for Health and Care Research (NIHR) Applied Research Collaborations (ARC) West Midlands; Office for Health Improvement and Disparities (OHID)—direct posts were also made to OHID regional and national health intelligence groups on an online platform for public service communication. Personnel at their West Midlands Knowledge and Intelligence Service (LKIS) were also contacted; Faculty of Public Health (FPH); and United Kingdom Public Health Register (UKPHR).

Survey respondents were directed to answer based on their most recent employment meeting the criteria and were asked to forward the survey to relevant colleagues. Reminders to complete the survey were sent in October 2023.

### 2.2 Comparative case studies

The primary data collection method was semi-structured interviews with key informants who met the previously stated eligibility criteria. Interviews used a guide to explore topics. The topics explored are available as supplementary information. Data was stored and organised in NVivo (15). An inductive thematic analysis of the interview data was performed (16). Firstly, for each topic, initial codes comprising meaningful segments of data—often the participants’ own words—were generated. Initial codes were then grouped into categories containing related codes and then, finally, categories were abstracted into themes. The product of this approach was a series of analytical codes, categories, and themes. Subsequently, a cross-case analysis was performed as described by Miles and Huberman (17). This involved identifying the commonalities and divergence between cases by cross tabulating the occurrence of categories within them. The cross-case analysis is available in the Open Science Framework data repository (18).

### 2.3 Survey of PHI staff

Survey questions were informed by case study themes. For example, a theme that emerged from case study interviews was the impact of pandemic response efforts on personal wellbeing. A survey question therefore sought to quantify this by exploring the degree of agreement with the statement “*COVID-19 negatively affected the wellbeing of staff working in PHI*” using a 5-point Likert scale (19). The survey included multiple choice, Likert Scale, and free-text questions and the topics explored are available as supplementary information. The Qualtrics platform was used to collect responses (20). The survey was piloted among case study participants and revised appropriately—it ran between 15th August 2023 and 20th November 2023. All quantitative questions were analysed using in-built features of the platform to produce descriptive statistics, e.g., the proportion of respondents in each Likert-scale category. Qualitative data from free-text responses was mapped by deductive coding to case study themes and then integrated with quantitative data as explained below. The survey analysis is available in the Open Science Framework data repository (18).

### 2.4 Data integration

Integration of qualitative and quantitative data was achieved in two ways. Firstly, as O’Cathain (21) describes, integration was built into the study design. This was achieved by using qualitative findings from the case studies to inform the topics explored quantitively via the survey. Secondly, after data from both collection methods had been analysed, qualitative and quantitative data was compared at the findings-level by the first author alone. As described by Fetters and Molina-Azorin (14), the meaning of findings as they related to each other was reflected upon and several possibilities considered including: confirmation—cause for drawing the same conclusion, complementarity—different but non-conflicting stories, expansion—provision of a broader but overlapping understanding, discordance—data conflict with each other. Quantitative data from the survey was compared with qualitative data sourced from the case studies and/or the free-text survey responses. These steps were checked by another researcher.

## 3 Results

Local authority (LA) case study sites included two metropolitan districts and two unitary authorities, representing both urban and rural populations. Twelve participants across the four case study sites were interviewed between November 2022 and June 2023. Table 1 details the case study sites.

**Table 1:** Descriptions of case studies.

Site code	LA type	Urban–rural classification^*^	Number of interview participants
A	Metropolitan district	Urban	3
B	Metropolitan district	Urban	5
C	Unitary	Rural	3
D	Unitary	Urban	1

^*^LAs were classified as urban or rural based on the 2011 rural–urban classification for LA districts in England (30). Sites were designated as either “rural” or “urban” if their classification incorporated these terms.

Participants were interviewed once and interviews lasted 1 to 1.5 h. Participants represented multiple levels of seniority: practitioner (*n* = 4), manager (*n* = 5), specialist/consultant (*n* = 2) and director (*n* = 1).

Data from 120 survey respondents was analysed. Table 2 shows respondent characteristics.

**Table 2:** Characteristics of survey respondents.

Characteristic	No. (%)
Worked for more than one English LA between January 2020 and present (*n* = 120)	24 (20%)
Gender (*n* = 115)	
Male	57 (50%)
Female	52 (45%)
Other	2 (2%)
Prefer not to say	4 (3%)
Age (*n* = 113)	
18–24	5 (4%)
25–34	16 (14%)
35–44	40 (35%)
45–54	29 (26%)
55–64	19 (17%)
65 and over	3 (3%)
Prefer not to say	1 (1%)
Ethnic group (*n* = 112)	
White	99 (88%)
Mixed	3 (3%)
Asian	4 (4%)
Black	0 (0%)
Other	1 (1%)
Prefer not to say	5 (5%)
Years of experience working in a PHI-related role (*n* = 110)	
Under 5 years	39 (35%)
5–10 years	30 (27%)
11 years and over	41 (37%)
Professional registration (*n* = 110)	
No registration	91 (83%)
Registration*	19 (17%)
UKPHR (practitioner)	5 (5%)
UKPHR (specialist)	10 (9%)
Others	6 (6%)
*Some respondents had more than one type of registration	
Type of LA (*n* = 109)	
Unitary authority	47 (43%)
County council	34 (31%)
Metropolitan borough	17 (16%)
London borough	9 (8%)
District council	1 (1%)
Town council	1 (1%)
Management responsibility (*n* = 109)	
Having management responsibility for others during work on COVID-19 response	55 (50%)

**Figure 2:**
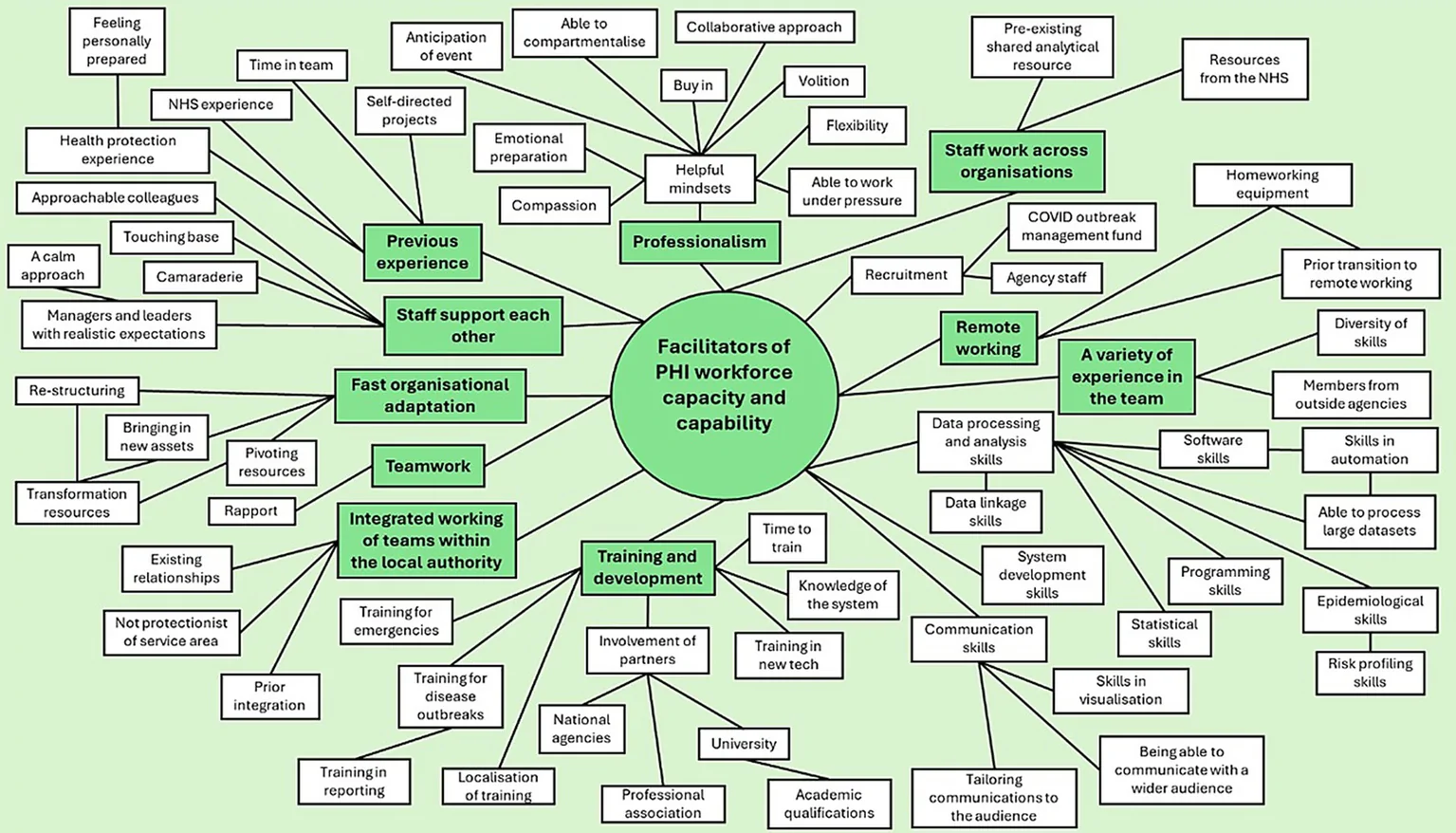
Workforce-related facilitators of local authorities’ capacity and capability to provide public health intelligence during the COVID-19 pandemic.

**Figure 3:**
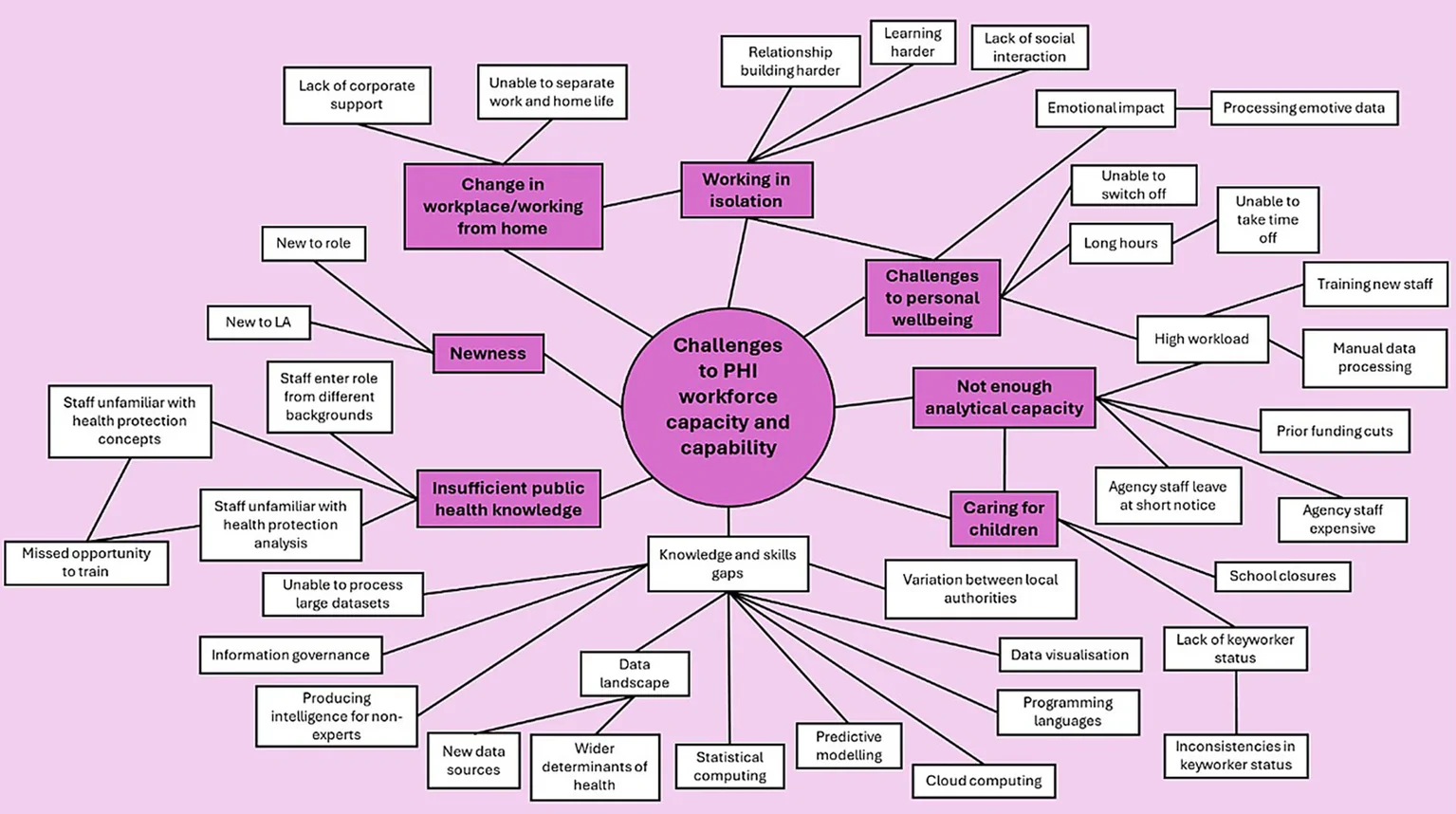
Workforce-related challenges to local authorities’ capacity and capability to provide public health intelligence during the COVID-19 pandemic.

The results of integrating case study and survey data relating to the capacity and capability of the PHI workforce to respond to COVID-19 are shown in Figures 2, 3. Prominent factors influencing responses, i.e., those categories occurring in interview data at more than one case study site, are shown in bold. The things that contributed to these (identified from all data sources) are also shown. The full analysis supporting these results can be found in the Open Science Framework data repository (18).

### 3.1 Facilitators of PHI workforce capacity and capability to respond to COVID-19

LA PHI staff involved in the response to the COVID-19 pandemic highlighted various workforce-related factors that facilitated their provision of PHI. Prominent categories of facilitator, i.e., those occurring at more than one case study site, together with contributing factors are outlined below.

Analytical capacity was boosted by fast organisational adaption, integrated working by LA teams, staff working across the health system and recruitment of temporary staff:

*“In addition to additional resource that we employed directly, we used resources from across the local health system—good analysts from providers who had an interest, for example, in predictive modelling.”* [Survey respondent]

And

*“Relied heavily upon colleagues across the organisation to assist.”* [Survey respondent]

When supported effectively by the LA, remote working facilitated response:

*“I felt the support colleagues received from my borough was good. Ranging from home working equipment, developing good working from home practices, latitude and understanding that people had to balance home commitments and work, wellbeing support and regular contact point.”* [Survey respondent]

Several factors supported staff’s wellbeing. At all case study sites these included teamwork and staff’s support for each other:

*“Having a fantastic team around me, helps me to do my job…having emotional support.”* [Participant 9]

In this context, case study participants described the significance of a good “environment” or “atmosphere”, having a sense of humour, being able to share frustrations, regular “catch ups” and feeling that others were approachable. The support of peers was also valued by survey respondents:

*“There was such comradery among everyone that it kept us going. And when someone had a wobble, there was a team of people to keep you going. I think ensuring strong team cohesion and ensuring nobody feels isolated is key.”* [Survey respondent]

Both case study participants and survey respondents referenced “Professionalism” as a key mindset that facilitated response. Related to this, flexibility was identified as a required attitude:

*“Be prepared for the unexpected at every single point and adapt to that.”* [Participant 8]

Case study participants also talked about needing to be able to work under pressure:

*“There had to be a willingness to get stuck in and be quite comfortable with things being vague but also quite time pressured.”* [Participant 11]

In addition, they thought the following behaviours were helpful: being able to compartmentalise, taking a collaborative approach, empathy and compassion. Survey respondents linked the following factors with feeling personally prepared: anticipating an event of this nature, being prepared emotionally, volition, and buy in.

A variety of experience in the team was a factor that PHI staff thought facilitated an effective response, for example, a survey respondent writes: *“there is value to maintaining a diverse workforce with diverse skillsets and knowledge of different tools and software.”* [Survey respondent].

Feelings of personal preparation were mediated by PHI staff’s skills and previous experience. For example, approximately a quarter of survey respondents (*n* = 27 or 28%) felt they were personally well prepared for the response. The reasons they gave included:

*“We were able to step up providing relevant reports on an automated basis and understood the relevant data landscape, information governance…”* [Survey respondent]

And

*“I have very good analytical and technical skills and was…easily able to pick up new datasets and link data from various data sources.”* [Survey respondent]

And

*“I'm used to looking at large datasets and death data.”* [Survey respondent]

Some respondents felt their qualifications were helpful:

*“My background was in epidemiology and I did a PhD previously to this role, so I was…able to answer questions we were being asked.”* [Survey respondent]

Participants at all case study sites reported needing communication skills. These included being able to tailor communications to the audience:

*“It was those skills in…understanding how to present my data and what sort of level of analysis…will be required for those sorts of groups…pulling out the key messages.”* [Participant 10]

And communicating to wider audiences:

*“More thought was given to how we visualise data given that there was this wider audience.”* [Participant 1]

At more than one site data processing and analysis skills, risk profiling, software skills (e.g., spreadsheets, mapping, data visualisation), and statistical and epidemiological skills were thought necessary.

Sometimes data systems were developed locally, and system development skills were useful:

*“R coding, infrastructure, and IT system development (e.g. had to implement the contact tracing system locally).”* [Survey respondent]

Interviewees at all sites thought prior health protection knowledge was beneficial, one person says:

*“Some of us who have worked in this field dealt with the 2009 H1N1 swine flu pandemic and that experience…was beneficial”* [Participant 5].

Training and development for PHI staff, both for disease outbreaks specifically, and in general, was seen as an important facilitator of response:

*“Local authority staff need to be trained in this work and be able to do it so they keep their skills live”* [Survey respondent]

At more than one site case study participants thought the following training should be provided to PHI staff to enable them to respond to similar events: epidemiology and statistics, health protection, knowledge of “the system”, new technology trends, and programming. Survey respondents felt skills in managing and analysing large data sets were desirable:

*“We need more staff skilled in managing and analysing large datasets in more automated, less intensive ways.”* [Survey respondent]

They pointed out the crucial role of skills in data visualisation:

*“I also had to learn Power BI very quickly it’s become a very useful skill.”* [Survey respondent]

And highlighted the value of training in reporting and understanding emergencies:

*“Having more training around reporting and understanding of emergencies.”* [Survey respondent]

Survey respondents’ suggestions on what should be included in training for PHI staff are detailed in Table 3.

**Table 3:** Survey respondents’ suggestions for elements to be incorporated in training for PHI staff.

Topic	Survey respondent quote
Preparedness	*“There should be much more focus on preparedness and specifically around these scenarios occurring.”*
Disease outbreaks	*“Roles may vary but I think should have some standardised training in disease outbreaks and relevant systems to get data and create dashboards etc.”*
Broader competencies	*“Psychology, social psychology, leadership and crisis management skills…”* and *“councils are incredibly political organisations (big and little p) any training should take this into account in terms of communicating and framing intelligence in terms of decision making in a political environment.”*
Analysis of wider data sets and research skills	*“The training that exists focusses more on analysis of NHS data but not on local authority or partner type datasets, or on knowledge management/research literature type skills.”*
Technological advances	*“The intel training available is very stats oriented, it’s a good base but does not account for technological developments of the last few years.”*

A greater proportion of survey respondents somewhat or strongly agreed with the statement *“Training for Public Health Intelligence staff should be more standardised”* than somewhat or strongly disagreed (*n* = 60 or 66% versus *n* = 8 or 9% respectively). Some were concerned about choosing a poor approach:

*“5 years ago the standard was Excel but this is a poor product compared to open-source programming languages.”* [Survey respondent]

The following quote encapsulates common themes:

*“There should be a universal training program with minimum standards available to all but there should also be an opportunity for all PH Intelligence staff to be trained in the local level too to understand how the key issues affect the local population. It would be beneficial to involve local partners in this too including primary and secondary care providers.”* [Survey respondent]

Some thought it desirable for staff working in local authorities and the NHS to have similar technical training and to know more about each other’s work:

*“…it’s keeping up to date with knowledge about NHS systems…if everyone accesses the same type of technical training on the data systems…it would be helpful…If people had that knowledge of what each other’s roles and responsibility and priorities are then it would help the system respond a lot more effectively.”* [Participant 12]

### 3.2 Challenges to PHI workforce capacity and capability to respond to COVID-19

PHI staff involved in the COVID-19 pandemic response identified several challenges they faced. Prominent categories of challenge, i.e., those occurring at more than one case study site, together with contributing factors are outlined below.

PHI staff at all case study sites expressed the view that there had been not enough analytical capacity. One participant said:

*“Our particular intelligence team was very small.”* [Participant 4]

Prior funding cuts were seen as a challenge:

*“The Public Health department at the time of the pandemic was underfunded and largely reduced in size.”* [Survey respondent]

However, more survey respondents somewhat or strongly agreed with the statement *“During the response to COVID-19 analytical resource was adequate in terms of people”* than somewhat or strongly disagreed with it (*n* = 53 or 55% vs. *n* = 36 or 37%). The most common response was ‘somewhat agree’ (*n* = 40 or 41%).

Open-ended survey responses may explain the discordance between the qualitative and quantitative data. When the survey was circulated time had elapsed. In many places more resource had been obtained. The response had also become less demanding and more efficient. Two quotes illustrate this:

*“The public health intelligence team needed to double in size in order to keep up with demand, and this did not happen for many months.”* [Survey respondent]

And

*“We were extremely overworked during the initial stages…As the requirement to set up new data flows ebbed, however, and the analytical requirements became more rote, the team capacity was sufficient.”* [Survey respondent]

Uncertainty around funding potentially delayed recruitment:

*“Initially there was the challenge of uncertainty on funding and inability to recruit until the funding was over the line.”* [Survey respondent]

Resource was often obtained from pausing other work:

*“All of PH Intel team was dedicated to only COVID from April 2020 to autumn 2021; all non-essential business as usual was postponed.”* [Survey respondent]

Agency staff were described as expensive, variable, and subject to leaving at short notice:

*“We did recruit additional capacity to meet requirements, but with this being through agencies it was expensive, variable in the people who filled the post, and uncertain times meant we had to replace people at short notice if, for example, they got permanent positions elsewhere.”* [Survey respondent]

Training new staff was described as an additional pressure:

*“Once the COVID Outbreak Management Fund came in, we could recruit and train up additional support, however, the training was an additional pressure.”* [Survey respondent]

Due to the closure of schools and childcare facilities, caring for children had been a challenge for some staff. Of those survey respondents who somewhat or strongly agreed their keyworker status during COVID-19 had influenced their ability to do their job (*n* = 29 or 30.9%), many said that the status had improved their access to schooling or care for their children. Inconsistencies in whether staff were conferred keyworker status were evident over time, between organisations, and within teams:

*“I did not initially have key worker status which meant my children could not go to school.”* [Survey respondent]

And

*“It was made quite clear to all of us that we were ‘not NHS’ and we were not treated as key workers.”* [Survey respondent]

And

*“Who said we were key workers? The consultants labelled themselves key workers and took advantage of school place availability, but not one single member of the Intelligence Team was offered any ‘key worker’ support. Ever.”* [Survey respondent]

Some commented that their ability to work from home meant that this status did not make much difference to them:

*“I don’t think that this made much difference. We were, perhaps, lucky that our employers had recently upgraded all our IT to laptops etc. so we could easily work from home”.* [Survey respondent]

Insufficient public health knowledge was a challenge to responding and half of survey respondents (*n* = 46) felt that COVID-19 had highlighted knowledge and skills gaps. Their comments indicate the need for skills in presentation and producing intelligence for non-experts:

*“To present data and intelligence to key council members and directors.”* [Survey respondent]

There was a view that the ability to process large volumes of data was lacking:

*“Handling and analysing large data.”* [Survey respondent]

There was a prominent theme about the need to automate data processing:

*“Local authorities can do more to improve IT infrastructure for automation/development of dashboards.”* [Survey respondent].

Predictive modelling was a skill in demand but sometimes missing:

*“Predictive modelling was wanted by everyone locally…we didn’t have the capacity (probably not the skills and definitely not the flexible, supportive approach) within our local authority to build the systems we needed.”* [Survey respondent]

Capability gaps in using software for data visualisation, cloud computing, statistical computing, and programming language for processing information in a relational database were often mentioned. Many survey respondents thought there was a knowledge gap around the data landscape:

*“Knowledge of where data is kept and who owns it.”* [Survey respondent]

This included data related to the wider determinants of health and from newer sources:

*“Data flows from care homes (care home tracker), Google data for movements, word searches etc., wastewater analysis etc., Generally using non-health/NHS data to gain insights.”* [Survey respondent]

And

*“Local economy impacts.”* [Survey respondent]

Expertise in information governance was also identified as a gap:

*“We relied on our information governance team to help us navigate the data sharing implications of outbreak management and using the line lists.”* [Survey respondent]

In contrast to these findings, participants across several case study sites indicated that they did not recognise any skills gaps.

There was some scope for improvement in terms of staff’s health protection knowledge and skills. Although, more survey respondents agreed or somewhat agreed with the statement: *“My health protection knowledge and skills were sufficient to respond effectively to COVID-19”* than disagreed or somewhat disagreed (*n* = 54 or 58% versus *n* = 24 or 26%), *n* = 40 or 43% of respondents answered “somewhat agree”. Some respondents were unfamiliar with how health protection was delivered:

*“I don’t think I knew a great deal…about how our health protection team functioned before COVID.”* [Survey respondent]

And/or unfamiliar with health protection concepts and data:

*“PHE introduced a number of concepts on the portal I had not been familiar with prior to the pandemic…e.g. infectious periods.”* [Survey respondent]

And

*“We’re working with data that we didn’t really fully understand.”* [Participant 10]

Qualitative and quantitative data on this topic complement each other offering different but non-conflicting stories. Survey respondents suggested there needed to be clarity around the LA role in health protection:

*“We need to be clear about whether health protection is in or out of scope for local authority analytical practice…and train people accordingly.”* [Survey respondent]

Survey responses revealed perceived challenges around training this group of staff including variation among local authorities:

*“The training and funding of public health intelligence teams varies greatly.”* [Survey respondent]

And staff with varying backgrounds enter the role:

*“Many people have [a] different background and [are] educated at different levels.”* [Survey respondent]

Newness, i.e., being new, either to the role or to a LA, was often given as a reason for not feeling well prepared:

*“I was new to public health and the local authority. Everything was new to me, not just the pandemic.”* [Survey respondent]

Across all sites, staff talked about challenges to personal wellbeing. It was evident from interviews that the response to COVID-19 was an emotive topic and had been traumatic for some. High workload and being unable to take time off were frequently mentioned by participants:

*“I was working probably seven days a week for a long period of time. So quite quickly, we faced burnout…and it does then affect your ability to respond when you’re just so tired.”* [Participant 9]

A survey question asked if respondents agreed with the statement *“COVID-19 negatively affected the wellbeing of staff working in PHI.”* The most common answer was “somewhat agree” (*n* = 39 or 40%). Far more respondents strongly agreed than strongly disagreed (*n* = 27 or 28% versus *n* = 1 or 1%).

A change in workplace/working from home challenged staff wellbeing in several ways. Participants mentioned not feeling able to separate work and home life:

*“It’s a very different world than what we were used to at the time. You’re not seeing people face to face…You are not having those little chats to get away from things. So, it’s very hard to separate…work and home-life.”* [Participant 4]

Lack of social interaction was also problematic for some:

*“Online working it’s been quite difficult, I think on the wellbeing of the team… especially people who are perhaps more in need of more social interaction.”* [Participant 12]

Another participant thought there was a lack of corporate support for homeworking:

*“What was missing for me was kind of a corporate view a corporate support on working from home... Obviously, there was from a very health and safety point of view ensuring the people have the right equipment, but not from a more kind of psychosocial point of view.”* [Participant 2]

The PHI role presented challenges including the need to process emotive data and not feeling able to switch off:

*“It was the emotional stuff as well…I was getting deaths data as well. Occasionally I saw people’s names there, that I know…And then, of course, out of work, it was on the news all the time. It was all anybody was talking about…So, there’s no getting away from it.”* [Participant 4]

For one person:

*“A lot of planning and common sense was an afterthought, for a profession that is well experienced on wellbeing and health protection management this was surprising.”* [Survey respondent]

Some respondents would have valued a calmer approach from senior staff:

*“At the beginning there was a lot of panic which resulted in unnecessary work/meetings/expectations. A calmer more considered approach would have been better.”* [Survey respondent]

Working in isolation caused difficulties in building relationships with people they had not met in person:

*“Building of partnerships and relationships with people who you hadn’t met.”* [Participant 2]

And in learning the job:

*“Learning a job, a new job for me…that’s really hard to do…from home. But normally, I’d be in an office and getting to know everyone…it was nothing but [a] barrier.”* [Participant 6]

## 4 Discussion

Our mixed methods study investigated the factors affecting public health intelligence (PHI) provision by English local authorities (LAs) during the COVID-19 pandemic. By exploring the views and experiences of PHI staff, we aimed to identify ways to enhance policy and practice to improve preparedness for similar events in the future. Interviews with 12 PHI staff involved in the COVID-19 response identified several key elements shaping it, including data infrastructure, public support, inter-organisational collaboration, leadership and management, and workforce capacity and capability. We conducted an anonymous online survey to explore these topics further and received responses from 120 PHI staff. Among the factors we identified as influencing response, the capacity and capability of the PHI workforce is a critically under-explored area, and this article therefore reports specifically on this aspect.

We found that many staff felt the available analytical resources, in terms of personnel, were insufficient at the start of the pandemic. Staff thought that factors contributing to this situation were prior funding cuts and a delay in recruitment caused by uncertainties around funding. As time went on, resources were brought in and the response became more efficient. Additional capacity was obtained via recruitment of agency staff, re-allocation of staff within the LA and the use of staff from across the health system. Key worker status seemed to make the most difference for people with childcare responsibilities as it facilitated access to schooling or care for their children. However, the data suggested that there were inconsistencies in who was conferred this status which may have caused inequitable access to these.

As well as analytical skills, many staff highlighted the need to be able to communicate well with a variety of audiences including non-experts in a political environment. Prior health protection knowledge was felt to be beneficial to staff, but some staff saw knowledge gaps around the health protection system and health protection data and analysis. Other potential gaps in knowledge and skills were in terms of being able to automate data processing and knowledge relating to information governance and the data landscape in terms of the wider determinants of health and newer data sources. In addition to formal knowledge, certain mindsets were viewed as helpful, such as anticipating an event of the nature of COVID-19 and being emotionally prepared. Training for staff was felt to be important for preparation - both in PHI generally - but also specifically for disease outbreaks. There was agreement that while a basic standard of training is desirable, diversity of skills within a team and localisation of training are also important. A perceived challenge was variation in training between local authorities, and some felt clarity around the LA role in health protection would help determine training requirements.

Many PHI staff viewed responding to the pandemic as having a negative effect on staff wellbeing and it was a traumatic time for some. Key factors influencing experience were high workload, inability to take leave, processing emotive data, and not feeling able to “switch off”. Homeworking presented challenges including some staff feeling they were more isolated and could not build relationships with colleagues (particularly those that were new to the role or to a LA). Staff’s support for one another and calm and supportive management and leadership were important mitigators of negative effects of responding to the pandemic on staff wellbeing.

Together, our findings reveal that at the onset of the pandemic, significant gaps existed in the capacity and capability of the PHI workforce, highlighting a shortfall in preparedness.

### 4.1 What this study adds

The findings from our primary research expand upon a mixed methods systematic review (MMSR) of the international literature on sub-national PHI responses to disease outbreaks we conducted as part of the broader project (22). While the MMSR also identified workforce capacity and capability to respond to infectious diseases as important, the primary data we collected offers additional insights. Notably, the MMSR did not include any studies from the United Kingdom and to our knowledge, this is the first United Kingdom study to explore the factors that influenced the provision of PHI during the COVID-19 pandemic from the perspective of LA staff, thus, addressing a gap in the existing literature. Our study offers the following new insights:

- The pandemic period was characterised by changing demands for analytical capacity and a mismatch between demand and supply in this respect.
- There were inequities in the allocation of keyworker status that disproportionately affected those with childcare responsibilities.
- LA PHI personnel perceived knowledge gaps around health protection, data automation, information governance, and new data sources.
- Pandemic response adversely affected staff wellbeing due to workload, leave restrictions, emotive data, and difficulty disconnecting.
- Staff viewed anticipating pandemics and emotional preparedness as valuable mindsets.
- Peer support and compassionate leadership played a crucial role in mitigating the negative impacts on wellbeing during the response.

Our findings underscore the need to:

#### 4.1.1 Prepare PHI staff for emergency response

Often LA PHI staff were not aware of emergency plans and did not feel prepared to respond to the COVID-19 pandemic. There is a legal duty to appropriately prepare staff for emergencies (23), and this is an issue that should be rectified with improved awareness and training around PHEP for PHI staff at all levels. To operationalise this, policymakers could direct that PHI staff receive annual emergency response awareness training and ensure that exercises with LRFs and health protection partners explicitly incorporate PHI functions. Policy makers should also review competency frameworks for public health staff, e.g., The Public Health Skills and Knowledge Framework (24), to ensure their content adequately encompasses learning from the COVID-19 response including from our and others’ studies.

#### 4.1.2 Implement best human resource practices

Employers of staff responding to an infectious disease emergency should follow best human resource practices. A key message is to consider how staff with different personal circumstances might be impacted by non-pharmaceutical interventions. Policymakers should provide clear guidance for employers of PHI staff on formal employment policies to support staff during emergencies. This guidance could encourage consideration of individual risk assessment, caring responsibilities, flexible or remote deployment, reasonable adjustments, rest and shift limits, occupational health referral, and manager wellbeing check-ins. Additionally, policymakers could advocate for pre-incident conversations with staff about potential constraints that may affect their deployment. Learning from other sectors should be drawn upon during guidance development.

#### 4.1.3 Improve public health workforce planning

There is a need to reduce inappropriate variation in staffing between local authorities and for baseline levels of PHI capacity and capability to be adequate. Policymakers can address this by enhancing data collection on the core public health workforce, including the PHI workforce, ensuring representation of all staff - not just public health specialists - and tracking key metrics such as human resource capacity, vacancy rates, turnover, skills mix, training, and emergency deployability. Benchmarking these metrics against similar authorities will help identify best practices and areas for improvement. Additionally, establishing a minimum PHI provision for each local authority footprint and developing shared capacity across organisations—while clearly distinguishing between core analytical capacity and surge capacity—will strengthen resilience. Clarifying roles and reporting lines in advance will further enhance preparedness.

### 4.2 Our results in context

Our findings complement research by Rotheram et al. (9) that examined local health protection responders’ experiences during the COVID-19 pandemic in England. Consistent with our observations, they found the response to the pandemic caused unprecedented disruption and workloads for staff. We draw upon their work to help explain our findings. They found inadequate capacity within local health protection systems resulted in PHE health protection teams transferring some tasks to LA staff. This underlines the importance of collective working and resources being used efficiently across the system. Unprecedented workloads and disruption during the COVID-19 pandemic may not have been unique to England. Sakai et al. (25) found public health workers in Japan were forced to work long hours and a need to respond to excessive workload, streamline operations, and to balance regular duties. They conclude it is important to be prepared to organise people flexibly during a pandemic and to anticipate that temporary support will be necessary.

The traumatic effect of the COVID-19 pandemic on some PHI staff might not have been anticipated in a group not considered ‘frontline’. However, this finding is consistent with other studies. Rotheram et al. (9) found that public health staff, like healthcare staff, experienced extreme stress during the pandemic and Singh et al. (26) found high levels of burnout in the Canadian public health workforce after 3 years of the COVID-19 pandemic (79%), with no significant differences by role. They found burnout positively associated with years of work experience, redeployment to the response, and not being offered workplace supports. Importantly, if the trauma we identified resulted in increased burnout among PHI staff, it may have had a profoundly negative impact on the profession and PHEP by undermining workforce capacity and capability. This would exacerbate existing challenges related to education and training, skills and competencies, staffing levels, service delivery, and career progression as identified by the CfWI (11).

### 4.3 Implications for policy and practice

Our results support the assertion of United Kingdom COVID-19 inquiry that there is a need to assess the practical capabilities and capacity to respond to emergencies: *“It is crucial when a pandemic starts to emerge to have access to up-to-date, comprehensive data about the United Kingdom’s response capabilities and capacity”* (2). Adequate staff resource, i.e., the right capacity and capability and contingency plans for staffing were suggested by staff as ways to prepare teams for making a similar response in the future. Staff highlighted the need for clarity on LAs’ role in local health protection. This is particularly important as there have been recent reorganisations of the health and care system in England that have led to increased fragmentation, i.e., the split of PHE into the OHID and UKHSA and the formation of ICSs. This division of responsibilities has implications for underpinning functions such as workforce development, data, and surveillance all of which affect the PHI workforce and PHEP and require careful consideration.

Our study highlights some of the key competencies PHI staff need to respond to infectious disease emergencies effectively, thus providing a foundation to develop targeted training to strengthen their response capabilities.

Our work emphasises the importance of supportive leadership and management during an infectious disease emergency. During the COVID-19 pandemic, PHI staff in English local authorities worked long hours for extended periods supporting crucial response work. They took on new areas of work and developed their knowledge and skills rapidly. However, the demands of response negatively impacted their well-being and some staff described feeling traumatised. It is therefore vitally important to consider and plan for workforce wellbeing during response to infectious disease outbreaks.

### 4.4 Strengths and limitations

This work stems from the first author’s 12 years of experience in PHI, including a managerial role within a local authority during the COVID-19 response. As a professional embedded in the field, their background directly informed the research focus. While their experience facilitated recruitment, data interpretation, and contextual understanding, it also introduced potential biases—such as social desirability bias during interview data collection (27). However, the exclusion of their former workplace as a case study site, adoption of data and methodological triangulation (including the use of an anonymous survey), systematic analysis, and the involvement of and oversight by other researchers with different professional backgrounds, may have mitigated this risk. The study thus reflects a balance between leveraging insider insights and ensuring methodological rigour.

Our mixed methods design helped us to develop a complete and rich understanding of the complex factors that influenced the COVID-19 response in the LA. Conducting multiple case studies allowed cross-case analysis meaning the research could move beyond a description of the response at each site, thus reinforcing its validity and generalisability and promoting theoretical elaboration (28). An inductive thematic analysis allows the data to “speak for itself” rather than imposing an existing framework (29). The integration of qualitative and quantitative data provides a more expansive understanding of the phenomenon than either alone. A strength of the research is that it captures the experiences and views of staff working at differing levels of seniority.

It is possible that LAs with more developed PHI functions were more likely to participate as case studies thus the case study findings may be skewed towards well-resourced and more engaged authorities so masking challenges faced by those that have less developed functions. No case study sites were two-tier authorities thus limiting the transferability of the results to this type of council. Both the previously mentioned concerns are mitigated by the survey data which includes responses from staff working in a range of LAs including two-tier authorities. A limitation of this study is the potential bias arising from the survey’s focus on a limited range of topics, as well as recruitment through professional networks and self-selection of participants. Furthermore, the uncertain size of the workforce prevented us from determining an exact denominator for the survey, which may limit the generalisability of our findings to the wider population of PHI staff.

## 5 Conclusion

Pandemics are inevitable, and our study has identified key priorities to ensure there is sufficient workforce capacity and capability to provide PHI effectively at a local level during such events. Our findings suggest that, in England, there is a need to improve public health intelligence workforce planning for emergencies. Emergency response plans should include the PHI function and not be divorced from the capacity and capability to enact them. In addition, the traumatic effect of responding to the COVID-19 pandemic on some PHI staff might not have been anticipated in a group not considered “frontline” workers. To mitigate these effects, we propose there is a duty to prepare staff for emergencies and implement best human resource practices. Improved PHI provision can guide public health actions that effectively reduce morbidity, mortality, and health inequities during future infectious disease emergencies.

## Data Availability

The datasets presented in this study can be found at: The Open Science Framework (https://osf.io/nmrt6/overview).
