# Supplementary material for: Enhancing public health intelligence workforce capacity and capability: insights from English local authorities’ COVID-19 response
Source: Front Public Health. 2026 Jul 1;14:1833664. doi: 10.3389/fpubh.2026.1833664 (PMC13369125; doi:10.3389/fpubh.2026.1833664)
Supplement: Supplementary file 1 [file Table_1.DOCX]

## Supplementary Material

Topics explored via the semi-structured interviews

| Interview topics |
| --- |
| - The structure used to deliver the COVID-19 response and how this evolved |
| - Data and information processed during the pandemic and the outputs produced from this |
| - Tasks performed |
| - Skills and tools utilised during the pandemic and how these differed to normal activity |
| - Challenges and facilitators to the response |
| - Viewpoints on preparation for COVID-19 |
| - Viewpoints on how to prepare for future disease outbreaks |

Topics explored by the survey

| Survey topics |
| --- |
| - Sufficiency of health protection knowledge and skills |
| - Resourcing in terms of people |
| - Staff wellbeing and work-life balance |
| - Use of software tools and whether this was optimal |
| - Disruption to and recovery of ‘business as usual’ |
| - Adequacy of debriefing/post-event review |
| - If standardisation of training is desirable |
